# Electronic cigarettes’ withdrawal severity symptoms among users during intermittent fasting: a cross-sectional study

**DOI:** 10.1186/s13722-021-00219-9

**Published:** 2021-02-05

**Authors:** Muna M. Barakat, Raja’a A. Al-Qudah, Ibrahim Alfayoumi, Hala Jehad Al-Obaidi, Feras Jassim Jirjees, Iman Basheti

**Affiliations:** 1 Faculty of Pharmacy, Applied Science Private University, Amman, Jordan; 2 School of Pharmacy, Queen’s University Belfast, Belfast, UK; 3 College of Pharmacy, University of Sharjah, Sharjah, UAE

**Keywords:** Electronic cigarettes, Smoking, Severity, Withdrawal, Physical, Psychological

## Abstract

**Background:**

Recently, electronic cigarette (e-cig) usage has increased significantly, making it a potentially effective smoking cessation tool. In Muslim countries, most people who use e-cigarettes fast the month of Ramadan, which results in intermittent fasting. This study aims to reveal the severity of e-cig withdrawal symptoms among users during this intermittent fasting period.

**Methods:**

A self-administered survey was developed and validated to solicit anonymous responses from e-cig users living in Jordan, through a cross-sectional study design. Participants were recruited through social media resources. Severity scores of physical (out of 11) and psychological (out of 8) withdrawal symptoms for each participant were assessed and calculated for each participant, depending on the symptoms reported.

**Results:**

A convenience sample (n = 523) of e-cig adult users were recruited. The majority of the participants were males (96.4%) aged between 18 and 40 years (86.4%). Many participants replaced tobacco smoking with e-cig (53.5%) in order to help them stop smoking. More than half of the participants experienced relatively weak physical (0.82 ± 1.78) and psychological (1.24 ± 1.89) withdrawal symptoms during the month of fasting. Most of the participants (63.2%) preferred to engage themselves with a busy schedule to tolerate the related withdrawal symptoms they experienced.

**Conclusion:**

E-cigs could play a vital role in smoking cessation among tobacco smokers during intermittent fasting. Ramadan offers a good opportunity for smokers to quit, as the reported physical and psychological e-cig withdrawal symptoms were found to be relatively weak. Awareness and behavioral interventions would help clarify the effect of e-cigs and help determine alternative ways to cease smoking.

## Background

Worldwide, electronic cigarettes (e-cig) usage has increased significantly in recent years, and it has been proposed as a potentially effective smoking cessation tool [1]. E-cig devices are defined as “a group of products that produce a heated aerosol, typically containing nicotine, which users inhale via a mouthpiece” [2]. E-cig usage has followed different patterns over the years, including dual use of tobacco and e-cigs among smokers, use by non-smoking youth, and young-adult smokers who have switched to the sole use of the e-cig [1]. Recently, the overall current prevalence of e-cig use in different countries has ranged from 2.8 to 3.2% [3], while, the prevalence of e-cig use in Jordan has been found to be much higher than that reported in other countries, ranging from 18 to 27.3%, with 50.9% replacing tobacco with e-cigs [4, 5]. According to a government study carried out by the Jordan health ministry in collaboration with the World Health Organization in 2019, 8 out of 10 Jordanian men smoke or regularly use nicotine products, including s-cigs [6]. With the exclusion of e-cig use and other smokeless patterns, research has shown that 66% of Jordanian men and more than 17% of women are smokers [6, 7].

Nicotine is widely known as a psychoactive drug and it is highly addictive [8]. Many nicotine smokers use smoking cessation products to stop smoking [8]. The main addictive component in many e-liquids used in e-cigs (referred to as vaping) is nicotine [1]. Thus, e-cigs can also lead to nicotine addiction for tobacco non-smoker adults and youth [1]. In addition, e-cigs may increase the severity of nicotine dependence in dual-users of cigarettes [1].

The long-term effects of e-cig use as a smoke cessation tool are debatable and have become a public health concern [9]. Furthermore, little is known about how vaping affects the use of and dependence on tobacco cigarettes, nor is much known regarding total nicotine use and dependence. Access to such information is vital when assessing the influence of vaping on smoke cessation and other health-related outcomes [10]. The onset, duration, and intensity of nicotine withdrawal symptoms depend on the smoking duration, the number of milliliters of the e-liquids consumed, and the concentration of the nicotine included. Hence, symptoms of withdrawal can last from several days to several weeks [10].

Different types of e-cig devices are currently available on the market, including the box mod, vape-pen, vape-pod and other different types [11]. E-cig devices have a specific e-liquid that is used to provide different concentrations of nicotine [12]. No previous study has looked into the severity of the withdrawal symptoms experienced by e-cig users in months of fasting. Ramadan is the holy fasting month for Muslims all around the world. Many things are prohibited during fasting time (from sunrise to sunset) including food, drink and smoking [13]. This month is known to be full of spirituality, religious worship and self-control [13]. During the month of Ramadan, people who follow the faith of Islam fast around 16 h a day. Ideally, this month is supposed to be a good chance for smokers to quit smoking [14]. Hence, this study is the first study conducted in Jordan that aims to evaluate the severity of e-cig withdrawal symptoms during intermittent fasting, particularly during Ramadan.

## Materials and methods

### Study design and participants

The study followed a descriptive cross-sectional design and the objectives were addressed via an online survey. The study was conducted in Jordan from 26 April to 15 June 2020. The online survey was developed and validated by clinical researchers to solicit anonymous responses, which were treated confidentially. Eligible participants included e-cig users who had experienced intermittent fasting, such as Ramadan. They could be of any nationality but must reside in Jordan. The inclusion criteria were explained upfront at the start of the survey (Additional file 1: Appendix S1). Participants were recruited through social media platforms: Facebook, WhatsApp, LinkedIn, and Twitter. Participants were also advised that their participation in the study was voluntary and that their participation did not pose any risks. Potential participants who completed the survey were considered to have given informed consent for participation.

### Survey development, validation, and reliability

The online survey was developed after reviewing similar validated surveys in the literature [15, 16] and was designed using the general principles of good survey design [17]. Several sources were used to generate a pool of questions considered to be relevant to the study objectives [15–18]. The online survey was finally prepared using the technology of Google Forms, and although it was constructed in English, it was delivered to the participants in Arabic—the formal language of Jordan. The survey contained multiple-choice questions and was designed to be completed within 10 min.

To ensure face validity, the first draft of the survey was evaluated by fifteen independent academics who had previous experience in smoking and vaping related work and research studies; a statistician was also involved at this stage of evaluation. The comments and feedback they provided were considered and incorporated where appropriate to prepare the final version of the survey. The survey was then translated from English into Arabic, and was then back-translated by two senior academic staff members who were considered fluent in both languages. Survey piloting took place to determine words that do not translate particularly well, words that are ambiguous, and whether or not another word or phrase would work better, and further, to confirm if the survey items were clearly presented. The questions were free from medical jargon or difficult terminology. To help the participants report accurate data, the popular types of e-cig devices were mentioned in the survey in the form of images, to help participants recognize the type of e-cig device they were using. Finally, the survey was piloted with a sample of 25 academic and 25 non-academic participants. This stage of the study was conducted to enhance clarity, readability, understandability, and confirm the study’s applicability to the Jordanian population. Internal consistency reliability was tested by the Cronbach’s alpha coefficient, which equaled to 0.88.

The final version of the survey contained three parts. The first part (Part A) comprised of nine questions, which included sociodemographic information; the second part (Part B) consisted of seven questions comprising details of participants’ use of e-cigs; the third part (Part C) consisted of 11 physical and 8 psychological withdrawal symptoms. The scores were assessed for each participant depending on the symptoms reported during the month of fasting, i.e. Ramadan. The participants were also asked to estimate the severity of withdrawal symptoms during the fasting month, using a score of severity (0–5). The severity reported by participants was rated as weak (score = 1) to very severe (score = 5). Participants were also asked to report the time their withdrawal symptoms started since their last e-cig use (in hours).

### Sample size

The most recent demographic statistics of citizens living in Jordan showed that 10.554 million people live in the country [19]. Based on that, the sample size was calculated using a margin of error of 5%, a confidence level of 95%, and a response distribution of 50%, giving a minimum sample size of 385 [20]. It was decided that the number should be increased to around 600 to take into account missing responses and other unknown issues that might arise.

### Statistical analyses

Completed surveys were extracted from Google Forms as an Excel sheet and were then exported to Statistical Package for Social Sciences version 24.0 (SPSS Inc., Chicago, IL, USA) for the statistical analysis. Descriptive statistics included percentages, means and frequency distribution, which were calculated for each question. Descriptive and univariate correlation analyses using Pearson correlation coefficient (r) were used for the correlation, which was conducted at a 5% significance level. A *p*-value of < 0.05 represented a significant difference. Factors affecting the severity scores of withdrawal symptoms were analyzed using simple and multivariant linear regression.

## Results

### Participant characteristics

#### Sociodemographic characteristics

Out of the total completed questionnaires (n = 610), eighty-seven forms (14.3%) were excluded from the main study measures due to missing data. Accordingly, 523 (85.7%) of the answered questionnaires were included in the study analysis. The majority of the participants were men (n = 504, 96.4%), and had a wide age-range with the highest percentage (48.2%) aged 26–40 years (Table 1). More than half of the participants (55.6%) were Jordanian in nationality and had a bachelor's university degree (n = 305, 58.3%). Only 7.6% (n = 40) of the study participants worked in the health sector, while others (n = 171, 32.7%) were employed in the non-health sector. More than half (n = 280, 53.5%) of the participants were single, while, (n = 241, 46.1%) were married and 41.3% had children.

**Table 1:** Sociodemographic characteristics of the participants (n = 523)

Characteristic	n (%)
Age	
Less than 18 years old	15 (2.9)
18–25 years old	200 (38.2)
26–40 years old	252 (48.2)
40–55 years old	55 (10.5)
Above 55 years old	1 (0.2)
Gender	
Male	504 (96.4)
Female	19 (3.6)
Education	
Didn’t complete school education	14 (2.7)
Secondary school certificate	114 (21.8)
Diploma	53 (10.1)
Bachelor's degree	305 (58.3)
Postgraduate degree	36 (6.9)
Occupation	
Secondary school student	41 (7.8)
University student	113 (21.6)
An employee in the health sector	40 (7.6)
An employee in a non-health sector	173 (33.1)
Housewife /Retired	7 (1.4)
Others	60 (11.5)
Social status	
Married	241 (46.1)
Single	280 (53.5)
Divorced/widowed	2 (0.4)
Having children	
Yes	216 (41.3)
No	27 (5.2)
Single and do not have children	280 (53.5)
Nationality	
Jordanian	291 (55.6)
Palestinian	11 (2.1)
Egyptian	80 (15.3)
Iraqi	109 (20.8)
Syrian	16 (3.1)
Lebanese	9 (1.7)
Other	6 (1.1)

#### Information related to e-cigarette usage

**Fig. 1:**
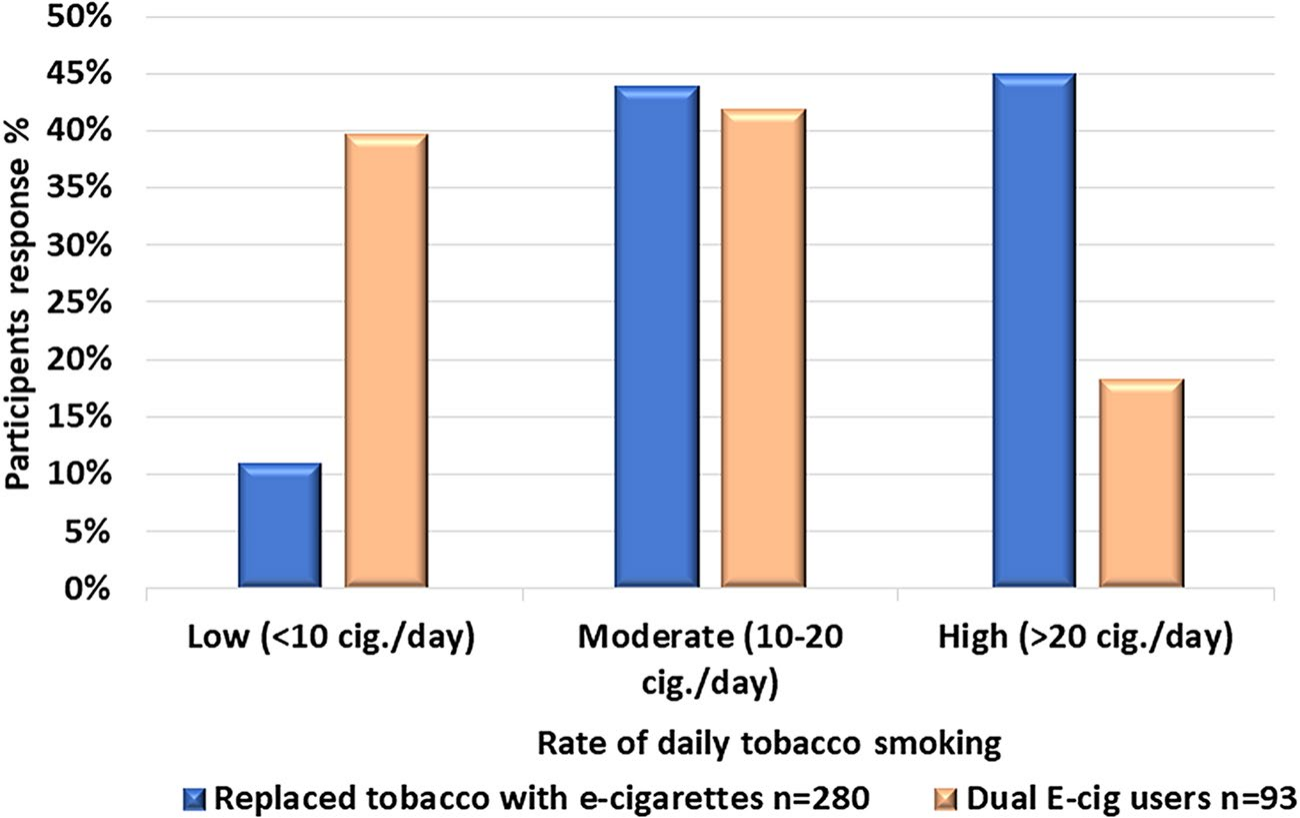
The percentage of E-cig users who replaced or combine tobacco smoking (n = 373)

Upon asking the study participants about the main reason behind their use of e-cigarettes, more than half (n = 297, 56.8%) reported smoking cessation as a reason (Table 2). Others (n = 157, 30.0%) stated that the e-cig has a better smell and taste than tobacco cigarettes. The percentage of participants who replaced tobacco smoking with e-cigs (n = 280, 53.5%) was significantly (*p* < 0.05) higher than both dual e-cig users (e-cig with tobacco smoking) and sole e-cig users. Many (n = 307, 58.6%) participants believed that the e-cig has the capability to reduce their desire for tobacco smoking and helped them cease tobacco smoking (Fig. 1).

**Table 2:** Basic information related to E-cigarette use among participants (n = 523)

Domain	n (%)
The reason for E-cigarettes usage	
To quit smoking	297 (56.8)
Love to try different new types of smoking	41 (7.8)
It has better smell and taste than tobacco	157 (30.0)
Easy to use	21 (4.0)
Safer than tobacco	7 (1.3)
The volume of consumed E-liquid per day	
Less than 2 mL	92 (17.6)
2–5 mL	283 (54.1)
More than 5 mL	148 (28.3)
Usage of E-cigarette concomitantly with tobacco	
Yes (dual E-cig users)	93 (17.8)
No, only e-cigarettes (sole E-cig users)	150 (28.7)
Replaced tobacco with e-cigarettes	280 (53.5)
E-cigarettes capability to reduce the desire for tobacco smoking and help to quit smoking	
Yes	307 (58.6)
Maybe	61 (11.7)
No	5 (1.0)
I don't smoke tobacco cigarettes, so I don't know	150 (28.7)

**Fig. 2:**
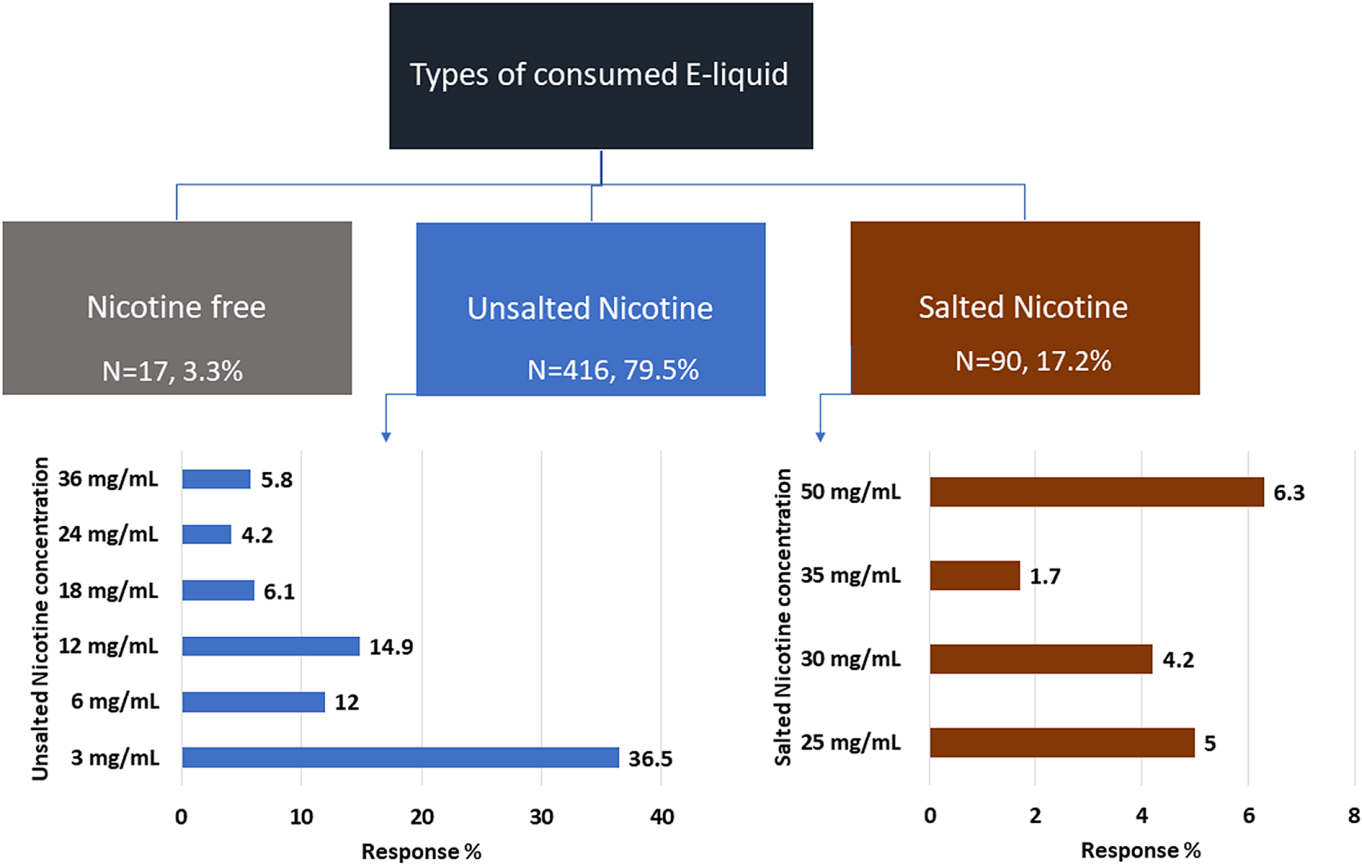
Summary for types of consumed E-liquids and the most common used concentrations of nicotine among the participants (n = 523)

The most commonly used e-cig device, as reported by the participants, was the ‘Box-mod’ (64.1%), followed by the ‘Vape pod’ (17.2%), then other types such as Ciga-like (10.9%) and ‘Vape pen’ (7.8%). More than half (54.1%) of the participants consumed between 2.0 to 5.0 mL/day of the e-liquid. The majority (n = 416, 79.5%) of the participants used unsalted nicotine-containing e-liquid (Fig. 2). The majority of those users (n = 191, 36.6%) consumed 2–5 mL/day of the 3 mg/mL nicotine-containing e-liquids. Hence, the daily nicotine consumption ranged between 6.0 and 15.0 mg/day. Other e-cig users (17.2%) used a salted nicotine-containing e-liquid, which was consumed using the ‘vape mod’ device.

#### E-cigarette withdrawal symptoms during fasting time

More than half of the e-cig users experienced both physical (55.1%) and psychological (53.0%) withdrawal symptoms during the fasting month of Ramadan. The most common physical withdrawal symptoms (Table 3) experienced by the participants included heart palpitations (10.3%), dizziness (10.5%), headaches (10.5%) and nausea (10.8%). Intense cravings (29.6%) and restlessness (22.5%) were the most common psychological withdrawal symptoms reported by the participants. Severity scores for both the physical (score out of 11) and psychological (score out of 8) withdrawal symptoms equaled 0.82 ± 1.78 and 1.24 ± 1.89, respectively. Those results indicated that most of the study participants experienced a relatively weak severity of withdrawal symptoms. Upon asking the participants to estimate the severity of withdrawal symptoms during fasting time using a score of severity (0–5, weak-sever) (Table 4), more than half (n = 288, 53%) of the e-cig users reported that the severity of withdrawal symptoms ranged between weak (score of 1, 20.1%) to very severe (score of 5, 0.8%). Some participants (15.9%; n = 81) reported that the withdrawal symptoms started in less than 6 h since their last e-cig use, while 15.5% (n = 81) started to feel the withdrawal symptoms after more than 16 h.

**Table 3:** Withdrawal symptoms experienced by the participants during fasting time (n = 523)

Physical symptoms (number of symptoms = 11)	n (%)	Psychological symptoms (n (number of symptoms = 8)	n (%)
No symptoms	235 (44.9)	No Symptoms	246 (47.0)
Weight gain	17 (3.2)	Social isolation	52 (9.9)
Sweating	22 (4.2)	Depression	56 (10.7)
Abdominal cramping	27 (5.2)	Feeling sleepy	60 (11.4)
Tremor	28 (5.4)	Poor concentration	77 (14.7)
Breathing Difficulty	36 (6.8)	Angry	94 (17.9)
Constipation	43 (8.2)	Stress	96 (18.3)
Sore or itchy throat	51 (9.7)	Restlessness	118 (22.5)
Heart palpitation	54 (10.3)	Intense cravings for smoking	155 (29.6)
Dizziness	55 (10.5)	-	
Headache	55 (10.5)	-	
Nausea	57 (10.8)	-	
Severity score Mean (± SD)	0.82 (± 1.78)		1.24 (± 1.89)

**Table 4:** Severity perception and onset of the E-cig withdrawal symptoms

Domain	n (%)
The severity of the withdrawal symptoms from the participants’ perception (range 0–5)	
No symptoms (score = 0)	246 (47.0)
Weak (score = 1)	105 (20.1)
Mild (score = 2)	84 (16.1)
Moderate (score = 3)	66 (12.6)
Severe (score = 4)	18 (3.4)
Very severe (score = 5)	4 (0.8)
Starting time of withdrawal symptoms from the last E-cig use	
In less than 6 h	83 (15.9)
After 6 h	31 (5.9)
After 8 h	43 (8.2)
After 12 h	39 (7.5)
After more than 16 h	81 (15.5)
I didn't experience any symptoms	246 (47.0)

Multivariable linear regression outcome (Table 5) showed a positive significant correlation (*p* ≤ 0.001) between the severity of physical and psychological withdrawal symptoms and the daily consumed nicotine concentration.

**Table 5:** Summary of the regression models for the dependent variable; Physical symptoms and psychological symptoms severity scores associated with participant’s characteristics, and nicotine concentration predictors’ usage

Withdrawal symptoms score	Physical withdrawal symptoms score				Psychological symptoms score			
	Linear regression		Multivariable regression		Linear regression		Multivariable regression	
	Beta	p-value	Beta	p-value	Beta	p-value	Beta	p-value
Gender, ref: Male	0.088	0.043*	0.089	0.042*	*− 0.004*	*0.929*	*NA*	*NA*
Age, ref: 26–40 years								
< 18 years	0.005	0.918	− 0.012	0.789	0.004	0.92	0.018	0.689
18–25 years	− 0.024	0.588	0.036	0.438	0.016	0.715	0.049	0.287
> 40 years	0.111	0.110	0.120	0.090	0.098	0.25	0.112	0.140
Level of education, ref: Bachelor's degree holder								
Didn’t complete school education	− 0.007	0.878	− 0.003	0.993	− 0.015	0.738	0.004	0.926
Secondary school certificate	0.079	0.07	0.074	0.104	0.091	0.037*	0.107	0.19
Diploma	− 0.094	0.031*	− 0.076	0.090	− 0.013	0.77	0.015	0.746
Postgraduate degree	0.053	0.225	0.057	0.203	0.072	0.102	0.089	0.051
Occupation: ref: employee in a non-health sector								
Secondary school student	− 0.017	0.691	− 0.026	0.579	0.027	0.538	0.032	0.495
University student	− 0.016	0.707	− 0.028	0.575	− 0.081	0.065	− 0.052	0.293
An employee in a health sector	− 0.029	0.508	− 0.037	0.434	0.026	0.547	0.031	0.502
Others	0.015	0.725	− 0.006	0.903	0.060	0.171	0.054	0.288
Social status, ref: married	− 0.159	0.309	− 0.079	0.356	− 0.370	0.032*	− 0.088	0.356
Having children, ref: yes	0.059	0.176	0.088	0.294	0.094	0.032*	0.032	0.294
Mean concentration of nicotine in E-liquid (14.71 ± 4.24) mg/ml	*0.021*	< 0.001*	*0.179*	< 0.001*	*0.136*	0.002*	*0.139*	0.001*

^*^significant value *p* < 0.05, *NA* not applicable

#### Participants responses upon experiencing e-cig withdrawal symptoms during fasting

Most of the participants (63.2%, n = 182) who suffered from withdrawal symptoms during the fasting month of Ramadan attempted to keep themselves busy to tolerate the withdrawal symptoms. Others (24.7%, n = 71) preferred to go back to tobacco smoking instead of e-cigs once they broke their fasting. Only 2.1% (n = 6) of the participants took advice from a pharmacist or other healthcare provider regarding their withdrawal symptoms. A small number of participants (1.4%) preferred using nicotine replacement therapy.

## Discussion

Globally, the prevalence of tobacco smoking increases greatly every year, which is associated with significantly high rates of mortality and morbidity [21]. In Jordan, recent statistics have shown that the tobacco smoking rate among the population is one of the highest rates in the world [22]. The use of e-cigs and their popularity has been well documented in the last few years worldwide. Specifically in Jordan, it is considered an innovative smoking tool that is sold as an alternative to traditional tobacco cigarettes. This cross-sectional study is the first to reveal the severity of e-cig related physical and psychological withdrawal symptoms during the fasting month of Ramadan among the general Jordanian population. Factors affecting these withdrawal symptoms have also been revealed. The majority of the participants were men who were using e-cigs for the purpose of smoking cessation. More than half of the e-cig users experienced physical and psychological withdrawal symptoms during intermittent fasting, particularly during Ramadan, including heart palpitations, dizziness, headaches, and nausea. However, the severity of these withdrawal symptoms was found to be relatively weak. A significant correlation was found between the severity of the physical and psychological withdrawal symptoms and daily consumed nicotine concentration.

Predictably, the majority of the study participants were men. Previous studies have shown a significant correlation between e-cig use and male gender due to different factors including smoking cessation, health care, and enjoyment purposes having a higher influence on men than women [4, 23]. Moreover, this finding could be related to the Jordanian cultural acceptance of public smoking for males only [24]. The usage of e-cigarettes among females, on the other hand, could be related more to recommendations from the surrounding milieu (family and friends), and psychological related causes such as stress and mood management [23].

Interestingly, the findings of this study showed that more than half of the e-cig users completely replaced tobacco smoking with e-cigs and were aiming to quit smoking via e-cig vaping. A study conducted by Berry et al. in 2019 showed that e-cig users were successful at quitting or reducing their tobacco smoking using e-cigarettes as a smoking cessation tool [25]. Besides, one-third of the participants chose e-cig use due to its better taste and smell compared to tobacco smoking. Piñeiro et al. also reported similar findings, as e-cig usage among men was found to be associated with positive expectancies, including better taste and social facilitation [23]. E-cigs are widely used for smoking cessation due to the common belief in their safety compared to tobacco smoking [25]. Similar results were found in a study conducted in Jordan, which showed that e-cig users agreed that using an e-cig is safer than tobacco smoking to both the user and surroundings, and that it is cheaper and can help in smoking cessation [4]. On the other hand, the literature suggests that high use of e-cigs is not medically recommended as most healthcare providers believe that e-cigs are unsafe and do not help with tobacco smoking cessation [26].

Ramadan is the holy fasting month for Muslims all around the world. Ideally, this month is supposed to be a good chance for smokers to quit smoking [14]. However, due to nicotine withdrawal symptoms, especially at the commencement of Ramadan, many fail to quit smoking [27]. Accordingly, smokers have reported following different methods to quit smoking, including a gradual reduction in nicotine consumption prior to fasting, using nicotine replacement therapy (particularly nicotine patches), and switching either completely or partially to e-cigs [14, 28, 29]. E-cig use yields quick and high levels of nicotine, which could help in smoke cessation, but conversely could also lead to physical dependence on nicotine [30]. A limited number of studies have investigated the occurrence and severity of nicotine withdrawal symptoms post the absence of e-cigs among users [30–32]. This study was concerned with this aspect during intermittent fasting, particularly during the month of Ramadan. Interestingly, more than half of the participants experienced relatively weak physical and psychological withdrawal symptoms, while the rest did not experience any withdrawal symptoms. Hughes and Callas state that a minority of e-cig users experience withdrawal symptoms when they quit or decrease the use of e-cigs, and suggest that the reported symptoms were much less than those reported by tobacco smokers [30]. Moreover, it was mentioned that e-cig withdrawal symptoms were not significantly greater in those who had attempted to quit e-cigs but failed. The importance behind revealing the severity of withdrawal symptoms comes from the fact that such severity can affect the cessation process [33]. In addition, the consequences of withdrawal symptoms, such as poor concentration or depression, could disturb the normal day of the life of the individual [34]. During fasting time, non-smokers usually complain of certain symptoms, such as headaches, dizziness and nausea, which could be related to hypoglycemia and/or caffeine withdrawal [35, 36]. In this study, only 10% of the participating e-cig users also reported symptoms that could be related to both fasting and nicotine withdrawal symptoms.

Intense craving (i.e. an increased urge to smoke) is one of the most common psychological withdrawal symptoms that contribute to smoking relapse and failure of cessation [37, 38]. In this study, around 30% of e-cig users suffered from intense craving during fasting time, which was associated with the usage of high concentrations of nicotine (24–50 mg/mL). Such findings could be considered a recidivism factor, which may limit the effectiveness of e-cig cessation during fasting and may require a strong and mindful will to succeed [38]. Development of coping behaviors and resilience processes during fasting (especially during Ramadan) could support e-cig users to quit and reduce the rate of smoking relapse [39–41]. Most of the study’s e-cig users preferred to keep themselves busy during fasting time as a way to help them control the withdrawal symptoms they experienced. This action could be useful in Ramadan, as this month is naturally rich with community gatherings and worship, which results in many distractions and thus empowers individuals to overcome physical and psychological withdrawal symptoms [27]. In addition, many published articles have highlighted the role of spirituality in the management and prevention of addiction (e.g. nicotine, alcohol, etc.) and have thus supported smoking or addiction recovery tools [42–44]. The minority of the study’s e-cig users reported that they preferred to take medications to manage their withdrawal symptoms, use patches of nicotine replacement therapy, and only a small number advised that they consulted a healthcare provider. Such responses highlight the suboptimal role of healthcare services offered to e-cig users, which are supposed to be essential for health consultations and user guidance in order to achieve the goal of smoke reduction and cessation [14]. It is important to note that the use of nicotine replacement therapy in the form of patches does not break the user’s fasting, as long as no direct oral intake happens [28].

More than half of the e-cig users consumed e-liquid containing nicotine (6.0 and 15.0 mg/day). There was a positive significant correlation between e-cig withdrawal symptoms and the daily consumed nicotine concentration. Most of the smoking-related studies demonstrated the addictive potential of nicotine, regardless of the source, be it tobacco smoking, waterpipe smoking, or e-cigs [2]. A study conducted by Talih et al. in 2015 found that higher nicotine concentrations in e-liquid and higher voltage in e-cig devices resulted in a higher nicotine yield via e-cigs [45]. However, the evidence has revealed that, in general, the nicotine produced from e-cigs is less than that from tobacco cigarette smoking [30]. The majority of this study’s participants used the ‘Vape mode’ device, which is a third-generation e-cig device that is characterized by providing the user the ability to change the voltage and the produced nicotine concentration [1]. Moreover, this study’s results show a significant correlation between the severity of withdrawal symptoms and the type of device being used.

The harmful effects of e-cigs are still not fully clear, and many cases have been recorded from around the world regarding the use of e-cigs, which include the induction of acute lung injury. There were documented cases for immature use of these devices as well, which contradict the original objective of their synthesis [46]. To summarise, this study’s findings indicate that e-cigarettes could play a successful role in smoking cessation among tobacco smokers via short term use by adults during intermittent fasting. Ramadan offers a good opportunity for smokers to quit. This should be managed under professional healthcare supervision and boosted by well-designed educational/awareness programs to facilitate the cessation during and post fasting. Further studies should be conducted to assess the applicability of such processes among e-cig users.

## Limitations

The first limitation of this study was the patient self-selection process that was followed. The survey was conducted online due to the novel coronavirus pandemic that started around January of this year, 2020 [47], and the public quarantine currently imposed in Jordan. Hence, only people who use the Internet and other social media platforms were able to participate. The second limitation is the representativeness of the sample to the population of Jordan, given that males actively participated in this survey more than females. The third limitation is that this study assessed the e-cig withdrawal symptoms among users during Ramadan only. Future studies should evaluate e-cig usage in months other than Ramadan, in order to compare the findings and provide a broader view of e-cig withdrawal symptoms. Moreover, some of the mentioned symptoms could be potentiated during fasting such as dizziness, headache and nausea.

## Conclusion

This study has shown that the majority of e-cig users sought smoking cessation as the main reason behind using e-cigs. The severity of the physical and psychological withdrawal symptoms upon the intermittent fasting during the month of Ramadan was relatively weak. Accordingly, these results could imply that e-cigs are compatible with being used as a smoking cessation tool in conjunction with intermittent fasting. However, success in ceasing smoking is highly dependent on the mindfulness of the Jordanian smoker and required motivational awareness programs and feasible behavioral interventions offered to support smoking cessation.

## Supplementary Information

**Additional file 1: Appendix S1.** Electronic cigarettes’ withdrawal severity symptoms among users during intermittent fasting survey.

## Data Availability

The data will be made available by the corresponding author upon request.

## Abbreviations

E-cig: Electronic cigarettes
E-liquid: Electronic liquid.
SPSS: Statistical Package for the Social Sciences
