# Supplementary material for: Electronic cigarettes’ withdrawal severity symptoms among users during intermittent fasting: a cross-sectional study
Source: Addict Sci Clin Pract. 2021 Feb 5;16:10. doi: 10.1186/s13722-021-00219-9 (PMC7864140; doi:10.1186/s13722-021-00219-9)
Supplement: Supplementary file 1 — Additional file 1: Appendix S1. Electronic cigarettes’ withdrawal severity symptoms among users during intermittent fasting survey. [file 13722_2021_219_MOESM1_ESM.docx]

**Electronic Cigarettes’ Withdrawal Severity Symptoms Among Users During Intermittent Fasting: A cross-sectional study**

We are a group of Jordanian researchers inviting you to fill this questionnaire. This study aims to investigate the severity of E-Cigarettes withdrawal symptoms during fasting time (especially during the month of Ramadan). It will be an observational study, subject to analysis and publication as a scientific piece of research. This means that your answers will be treated in strict confidence.

The questionnaire consists of three sections, which will require 7-10 minutes of your time to answer it, knowing that the questionnaire does not require writing the name or any other private information.

If you are an E-cigarette user, fasting during Ramadan and living in Jordan. your participation in completing this questionnaire is highly appreciated

Agree to participate

Disagree to participate

(**Basic information (Demographic**

- **Gender**

Female

Male

- **Age**

Less than 18 years old

18-25 years old

26-40 years old

40-55 years old

Above 55 years old

- **The highest education level**

I didn't complete school education

Secondary school certificate

Diploma

Bachelor's degree

Postgraduate degree

- **Occupation**

Secondary school student

University student

Housewife

An employee in the health sector

An employee in a non-health sector

Retired

Others

- **Social status**

Single

Married

Divorced

Widow

- **Do you have children?**

Yes

No

I am single and I do not have children

- **Nationality**

Jordanian

Palestinian

Egyptian

Iraqi

Syrian

Lebanese

Other

- **If you are a tobacco smoker (current or former), how long did you smoke?**

I never smoke

5 years or less

More than 5 years

- **How do you rate the degree of your smoking (current or former)?**

Low (less than 10 cigarettes per day

Moderate (between 10 to 20 cigarettes per day

High (more than 20 cigarettes per day)

**Basic information related to E-cigarette use**

- **Why did you start using E-cigarettes**?

To quit smoking

I love to try different new types of smoking

It has a better smell and taste than tobacco

Easy to use

Safer than tobacco

- **Do you use nicotine-containing E-liquids?**

Yes

No, I use free nicotine E-liquids

- **What is the concentration of nicotine that you use?**

zero mg/mL (if you use nicotine free e-cigarettes)

3mg/mL

6 mg/mL

12 mg/mL

18mg/mL

24 mg/mL

36 mg/mL

25 mg/mL (salted nicotine)

30 mg/mL (salted nicotine)

35 mg/mL (salted nicotine)

50 mg/mL (salted nicotine)

- **How many milliliters of E-liquid do you usually consume per day?**

Less than 2 mL

2-5 mL

More than 5 mL

- **What is the type of E-cigarettes you used to use?**
- Box-mod
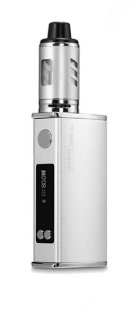

- Vape-pen
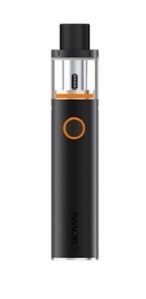

- Vape pod
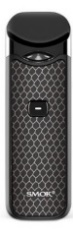

- Others
- **Do you smoke both types of cigarettes (tobacco and electronic) at the same time?**

Yes

No, only e-cigarettes

No, I replaced tobacco with e-cigarettes

- **Do you feel that E-cigarettes can reduce your desire for tobacco smoking and help you quit smoking?**

Yes

Maybe

No

I don't smoke tobacco cigarettes so I don't know

**Information related to E-cigarette Withdrawal symptoms**

- **Have you ever experienced any withdrawal symptoms during Fasting or after quitting E-cigarettes?**

Yes

No

- **Which of the following symptoms (the physical withdrawal symptoms) did you experience the most during fasting time?**

Sweating

Heart palpitation

Breathing Difficulty

Sore or itchy throat

Tremor

Abdominal cramping

Nausea

Dizziness

Headache

Constipation

Weight gain

I didn't experience any symptom

- **Which of the following symptoms (the psychological withdrawal symptoms) did you experience the most during fasting time?**

Intense cravings for smoking

Restlessness

Poor concentration

Stress

Depression

Social isolation

Feeling sleepy

Angry

I didn't experience any symptom

- **What did you do to alleviate these symptoms? (you can choose more than one answer)**

Taking medications to manage the symptoms

Using nicotine replacement therapy (patch)

Taking advice (counseling) from a pharmacist or medical health staff

keeping my self-busy to tolerate those withdrawal symptoms

Getting back to tobacco smoking instead of E-cigarette

I didn't experience any symptom

- **Please Score the severity of the general withdrawal symptoms that you have experienced during fasting time (from 0 to 5)**

0 (if there weren't any symptoms)

1 (weak)

2 (mild)

3 (moderate)

4 (severe)

5 (very severe)

- **Usually, your withdrawal symptoms start to appear (during fasting time)**

In less than 6 hours from your last smoking.

After 6 hours from your last smoking.

After 8 hours from your last smoking

After 12 hours from your last smoking.

After more than 16 hours from your last smoking

I didn't experience any symptom
